# Modification of Ti-Al-V Alloys with Layers Containing TiN Particles Obtained via the Electrophoretic Deposition Process: Surface and Structural Properties

**DOI:** 10.3390/ma17235710

**Published:** 2024-11-22

**Authors:** Maria Biegun-Żurowska, Anna Berezicka, Marcin Gajek, Tomasz Goryczka, Magdalena Ziąbka

**Affiliations:** 1 Department of Ceramics and Refractories, Faculty of Materials Science and Ceramics, AGH University of Krakow, 30-059 Krakow, Poland; berezicka@agh.edu.pl (A.B.); mgajek@agh.edu.pl (M.G.); 2 Institute of Materials Science, University of Silesia in Katowice, 75 Pułku Piechoty 1A, 41-500 Chorzow, Poland; tomasz.goryczka@us.edu.pl

**Keywords:** titanium alloy, titanium nitride particles, chitosan EPD, electrophoretic deposition, surface and structural properties

## Abstract

The aim of this work was to obtain homogenous coatings containing chitosan with different concentrations of titanium nitride particles (TiN). The coatings were deposited via an electrophoretic process on an etched medically pure Ti-6Al-4V alloy. As part of the study, the zeta potential of the suspensions used for EPD coating deposition was measured, allowing for the optimization of process parameters and the assessment of suspension stability. Subsequently, the research focused on evaluating the microstructure (SEM + EDS), structure (XRD), and surface characteristics (roughness, contact angle, surface energy, microhardness, coating adhesion) of the deposited layers. SEM microscopy confirmed the effective deposition of titanium nitride particles onto the titanium alloy surface. XRD analysis proved the assumed phase composition of the coating. The increase in TiN phase content in the individual layers was confirmed. The chitosan/TiN layer’s introduction altered the alloy surface, increasing its roughness and static water contact angle. The highest roughness and hydrophobic properties were observed in the coating with a 2 wt.% concentration of titanium nitride particles. Additionally, the coating containing the highest concentration of ceramic particles (2 wt.%) exhibited the highest hardness (197 HV) among all the tested layers. However, the TiN particles incorporation in the layer decreased the adhesion strength, from 2.36 MPa (0.5 wt.% TiN) to 1.04 MPa (2 wt.% TiN). The coatings surface and structural properties demonstrate potential as protective layers for implants and are suitable for further biological studies to assess their applicability in medical and veterinary fields.

## 1. Introduction

In recent years, due to the changes in lifestyle together with increasing population ageing [1], biomaterials have witnessed a significant demand in research and innovation aimed at enhancing the performance and biocompatibility of medical implants [2,3,4]. Over the last years, veterinary medicine has also changed its approach to animals’ osseous injuries. Nowadays, bone implantation is used not only in humans bone injuries, but also veterinary surgeons are employing different types of implants during treatment procedures [5,6,7].

One of the most commonly used metallic material for bone implantation are biocompatible titanium alloys [8,9,10]. Titanium and its alloys stand out as prominent candidates for orthopedic implants due to their remarkable mechanical properties, corrosion resistance as well as the satisfactory bio-tolerance [4,11]. Lack of toxicity and low elastic modulus are significant parameters in the development of biocompatible titanium alloys [4,8].

The two-phase medical Ti-6Al-4V titanium alloy boasts exceptional characteristics, including high strength, low density, superior fracture toughness, and outstanding corrosion resistance [12,13]. Grade 5 titanium alloys containing appropriate amounts of titanium, aluminum and vanadium are characterized by appreciable mechanical properties which are desired in the case of implantation. However, the presence of vanadium and aluminum in the alloy composition increases toxicity and might slow down the Ti-Al-V implant osseointegration [9,14].

Despite the high biocompatibility of medical titanium alloys, their implantation can cause adverse reactions in the surrounding tissues [1,11]. Implant placement in a living organism carries risks of unfavorable complications, such as ions release [14], microbial infections [11], collagen formation or even necrosis [7,9,14]. In order to mitigate these implant-related negative effects, scientists are constantly improving mechanical and biological properties of metallic implants. One of the solutions is to enhance the implant surface with a properly designed multifunctional coating [9] to ensure antibacterial properties and protect it against corrosion or scratches. These requirements can be achieved by applying an appropriate concentration of antibacterial agents, e.g., different types of nanoparticles, in the coating composition [1,2].

There are numerous surface engineering methods for depositing coatings with an appropriately selected composition and properties [15,16,17]. Each of the available methods has different advantages and limitations [3,9,17]. The selection of the appropriate coating system and deposition method is a complicated process, dependent on its application, compatibility of the coated materials, substrate surface and shape, available equipment as well as deposition parameters and process costs [18].

In this study, coatings were deposited utilizing the electrophoretic deposition (EPD) method which stands out as a specialized colloidal processing technique. It capitalizes on the charged particles migration in a solution driven by an electric field, ensuring their methodical deposition onto a substrate [19]. Similarly to each of the available methods, EPD technique displays some limitations. The first significant disadvantage of EPD is that it is not preferable to use water as the dispersive phase. This is due to the electrolysis process, which produces hydrogen and oxygen gases, adversely affecting the quality of the deposited coatings. Another drawback of EPD for ceramic coatings is their poor adhesion to metallic substrates, often necessitating additional heat treatment of the deposited layers [20]. Furthermore, the homogeneity of the deposited coatings heavily depends on the electrical conductivity of the substrate, limiting the range of suitable substrate materials [20,21].

Nevertheless, the electrophoretic deposition (EPD) method offers a multitude of advantages, including suitability for simultaneous co-deposition of various materials [22], the possibility to perform the deposition process at room temperature [22], the capacity to conform to intricate substrate shapes. The EPD process provides the flexibility to apply coatings on diverse materials with varying shapes and sizes, leading to the flawless deposition of coatings ranging from sub-micron to millimeter dimensions [23]. Moreover, the deposition process is both controllable and repeatable [24]. Technical benefits of EPD include the simplicity of equipment and a short deposition time [24].

Due to its many advantages, the electrophoretic deposition technique is currently attracting attention as the effective solution for processing bioactive coatings and biomedical nanostructures [19]. One of the key challenges in this field is optimizing the composition of EPD coatings to enhance both their mechanical and biological properties. Titanium nitride (TiN) and its derivatives are particularly promising materials for implant protective coatings, owing to their high hardness, excellent wear and scratch resistance, favorable wettability, low coefficient of friction, and physiological inertness [25,26]. Studies suggest that the deposition of titanium nitride coatings on orthopedic implants can have a positive impact on the biocompatibility and tribological properties of bone implant surfaces [27]. Furthermore, TiN is known for its low toxicity in both in vitro and in vivo environments [26], and is being explored as a potential antibacterial agent [28,29]. The use of titanium nitride in the form of particles is also widely discussed as an alternative material for various biomedical applications [30,31].

The primary objective of this study was to investigate how the addition of varying amounts of TiN particles to chitosan-based coatings influences the microstructure, chemical composition, and surface properties of coatings deposited on a Ti-Al-V alloy. We focused on developing coatings with strong adhesion and robust mechanical properties, aiming to create a protective and antibacterial layer for implants. The initial step involved producing durable, well-adhered layers with optimal mechanical characteristics to protect implants from scratches and other forms of damage. Furthermore, the antibacterial potential of TiN will be examined in subsequent biological testing, as this feature is crucial for preventing infections and enhancing the longevity of biomedical implants. By enhancing specific surface and mechanical attributes, our study addresses key factors in improving the overall performance of biomedical coatings, while highlighting the promising antibacterial capabilities of TiN for future biomedical applications.

## 2. Materials and Methods

Metallic substrates (2.5 × 1.5 cm plates) made from grade 5 Ti-6Al-4V alloy were provided by Wolften (Wrocław, Poland). Coatings were applied to the Ti-6Al-4V plates using the electrophoretic deposition (EPD) technique. Titanium nitride (TiN) particles, with an average size of less than 3 μm and a BET surface area of 2.6142 ± 0.1102 m^2^/g, were sourced from Sigma-Aldrich (Taufkirchen, Germany).

To prepare the final EPD suspensions, five different suspensions with TiN mass concentrations of 0%, 0.5%, 1%, 1.5%, and 2% were created. Each of these final suspensions was formed by combining two initial suspensions. One suspension contained 0.125 g of medium molecular weight chitosan (Sigma-Aldrich, Germany) dissolved in a mixture of 20 mL distilled water and 0.9 mL acetic acid (Pureland, Gliwice, Poland). The other suspension contained varying amounts of TiN particles to achieve the desired final concentrations, along with 5 mL of isopropyl alcohol (Pureland, Poland) and 25 mL of ethanol (Poch, Gliwice, Poland).

Both initial suspensions were first subjected to ultrasonic agitation for 1 h. Having mixed the two suspensions, the obtained combined solution underwent an additional hour of ultrasonic agitation, followed by 24 h of magnetic stirring. Just before the deposition process, the final suspension was ultrasonically agitated for 2 min.

The zeta potential of the prepared solutions was measured using a Zetasizer Nano-ZS apparatus (Malvern Instruments Ltd., Malvern, UK), applying the Smoluchowski equation for calculation [32]. A 0.01 M NaCl aqueous solution was used as the background electrolyte.

Prior to EPD, the titanium alloy plates were ultrasonically cleaned in acetone (Pureland, Poland) for 30 min, followed by a 30 min ultrasonic treatment in ethanol (Poch, Poland). Just before deposition, the plates were etched in a 5% hydrofluoric acid solution (Chempur, Piekary Śląskie, Poland) for 30 s and rinsed with distilled water. The electrophoretic deposition was carried out at 30 V for 3 min, with the Ti-6Al-4V alloy acting as the cathode and a stainless-steel electrode as the anode. The distance between the electrodes was maintained at 1 cm.

**Figure 1:**
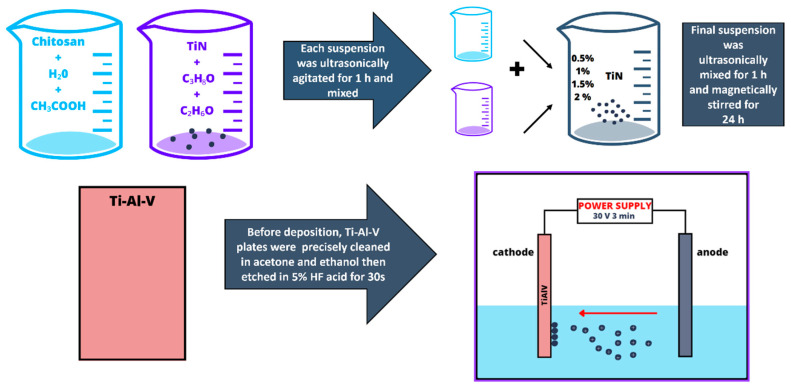
Solution preparation and EPD process scheme.

A schematic of the solution preparation and EPD process is shown in Figure 1. Table 1 provides the standardized nomenclature used for naming the samples in this study.

### 2.1. Structural Studies

The SEM-EDS investigations were conducted utilizing the Apreo 2S high-resolution scanning electron microscope from ThermoFisher Scientific (Waltham, MA, USA). Depending on the sample, observations were made with the in-column detector T1, low vacuum detector LVD or Everhart–Thornley Detector (ETD) at an accelerating voltage of 5–10 KV. Qualitative and quantitative analyses were carried out through a standardless method employing the SDD detector, specifically the EDAX Octane Elite with APEX™ Advanced 2022 software, version 2.5.1001.0001.

The X-ray diffraction (XRD) patterns were measured using the X’Pert Pro PANalytical diffractometer (Malvern Panalytical Ltd., Malvern, UK). This instrument was equipped with a copper lamp emitting monochromatic radiation with the wavelengths of 0.1530598 nm (CuKα1 line) and 0.1544938 nm (CuKα2 line) and the GIXD attachment. Diffraction patterns were measured in the classical Bragg–Brentano (BB) geometry at the 2θ range 5–140°, whereas in GIXD mode from 5 to 100°. The step was 0.05° with the counting time adjusted to receive proper counting statistics.

### 2.2. Surface Properties Studies

The samples surface topography was observed using the laser confocal microscope Lext OLS 4000 (Olympus, Tokyo, Japan). The scanned area was 256 × 256 μm.

The arithmetical mean roughness (Ra) and root mean square roughness (Rq) of the examined coatings were determined utilizing the HOMMEL-ETAMIC T1000 wave contact profilometer (Jenoptik AG, Jena, Germany). The presented values were computed as an average of 5 measurements and the results are presented as the mean ± standard deviation SD.

To assess the surface wettability of the samples, the static water contact angle (WCA) was used. Wettability was measured via the sessile drop method with the automatic drop shape analysis system DSA 10 Mk2 (Kruss GmbH, Hamburg, Germany). The measurement was performed in the constant temperature and humidity conditions. Each UHQ-water droplet with the 1 μL volume was applied on a dry and clean specimen. The contact angle was calculated as an average of 25 measurements, the results are presented as the mean ± standard deviation SD.

The assessment of free surface energy involved testing contact angles with two types of measuring liquids: ultra-pure distilled water (UHQ PURE Lab, Vivendi Water, Paris, France) and diiodomethane. The measurements were conducted at room temperature using the DSA 10 Mk2 optical apparatus from Kruss GmbH, Hamburg, Germany. The free surface energy was determined based on the dispersion and polar components derived from the obtained photos of the two measuring liquids drops. The reported value represents the arithmetic average from 25 measurements and the results are presented with the corresponding standard deviations (SD).

### 2.3. Mechanical Studies

To evaluate the microhardness of the EPD chitosan/TiN coatings, Vickers microhardness measurements were conducted using the FM700 hardness testing machine (Future Tech, Corp., New York, NY, USA) with an applied load of 0.25 N. The experiments took place at room temperature under atmospheric conditions, and the reported value represents an arithmetic average of 10 measurements. The results are expressed as the mean ± standard deviation (SD).

The adhesion strength values were determined on the basis of tests carried out using the Zwick-Roller testing machine (Ulm, Germany). The tensile strength was measured (test speed of 2 mm/min), in accordance with PN-EN ISO 4624:2023-11 [33] on 4 samples of each material. The results are expressed as the mean ± standard deviation (SD).

### 2.4. Statistical Analysis

Statistical analysis of the results was performed using the one-way analysis of variance (ANOVA) accompanied by Duncan’s post hoc tests, performed using the Statistica 13.3 software (TIBCO Software Inc., Palo Alto, CA, USA). The results were deemed statistically significant if *p* < 0.05.

## 3. Results and Discussion

**Figure 2:**
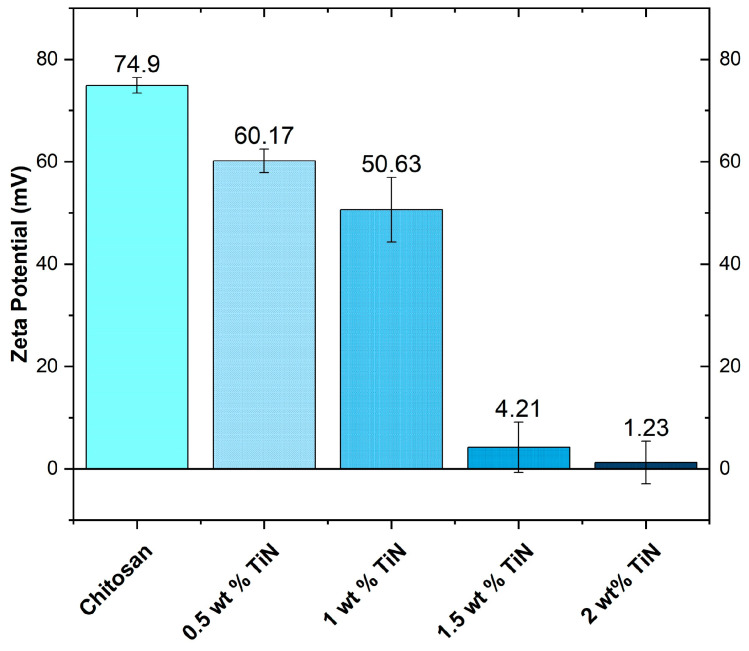
Zeta potential of the chitosan-TiN EPD solutions.

The zeta potential analysis was conducted prior to initiating the electrophoretic deposition process to assess the stability of solutions containing chitosan and varying quantities of titanium nitride particles. The zeta potential values for the chitosan/TiN solutions are shown in Figure 2. As the particle’s quantity increased, the zeta potential of the prepared chitosan-TiN solutions gradually decreased, ranging from 74.9 mV (0 wt.% TiN) to 1.23 mV (2 wt.% TiN). It is important to emphasize that there are several possible explanations for this phenomenon, which include particle agglomeration, where aggregated particles can alter the effective surface charge of the bath, resulting in different zeta potential values. Additionally, chitosan interaction may play a role, as chitosan could interact differently with TiN particles at varying concentrations, thereby impacting its distribution and behavior within the plating bath, and consequently affecting zeta potential values. Moreover, electrochemical effects might be at play, as variations in TiN concentration can influence electrochemical processes within the plating bath, known to affect zeta potential by altering the distribution of charged species and surface charge properties. However, based on the authors’ knowledge, the most probable factor responsible for these differences is the differing TiN concentration itself. Citing the DLVO theory [34], it is acknowledged that van der Waals forces and repulsive electrostatic forces are the primary interactions between TiN particles in suspension. The potential energy of these interactions is influenced by the concentration of TiN particles present. Higher concentrations of TiN particles can lead to a closer packing within the suspension, affecting the balance of these forces and potentially reducing electrostatic repulsion between particles [35,36].

**Figure 3:**
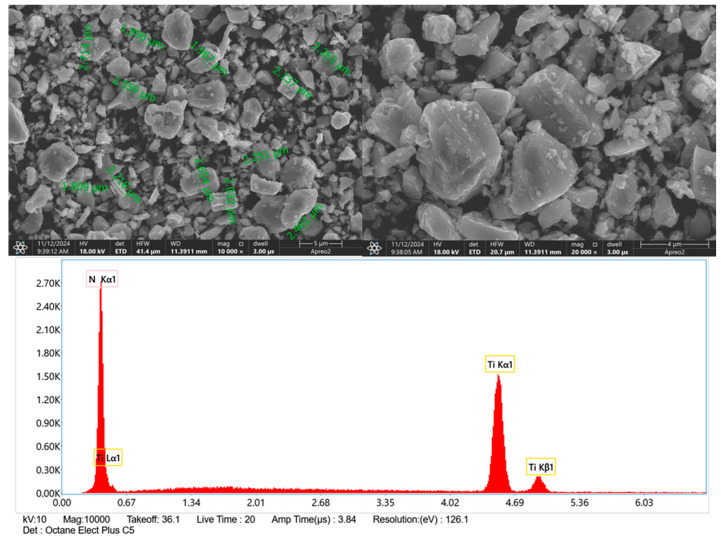
SEM images and EDS spectra with element content of the titanium nitride particles, used in the experiment.

The SEM images of the titanium nitride particles were taken prior to deposition to assess their morphology, shape, and size (Figure 3). The particles were found to have an approximate size of less than 3 μm, with sizes ranging from approximately 1.96 μm to 2.71 μm, as shown in the annotated measurements. The particles exhibited irregular, angular shapes with rough and uneven surfaces. Following the SEM evaluation, Energy Dispersive X-ray Spectroscopy (EDS) was performed. The EDS spectrum confirmed the presence of only two elements: titanium (Ti) and nitrogen (N). This indicated the purity of the particles as titanium nitride (TiN) with no detectable impurities. The distinct peaks for nitrogen (N Kα1) and titanium (Ti Lα1, Ti Kα1, and Ti Kβ1) in the spectrum corroborated the composition of the TiN particles.

**Figure 4:**
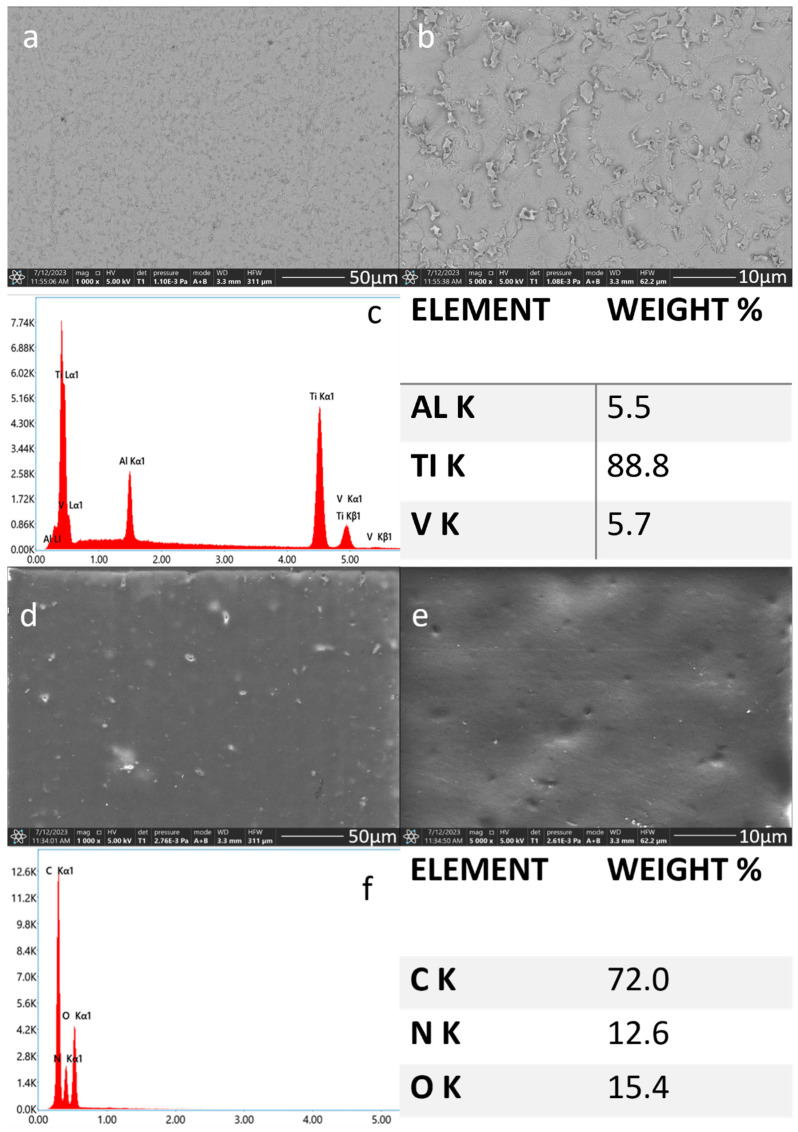
SEM images and EDS spectra with element content of the titanium alloy (Ti-Al-V) etched with HF (**a**–**c**) and covered with chitosan (**d**–**f**).

The scanning electron microscopy observations confirmed the successful formation of EPD coatings with increasing amounts of titanium nitride particles, deposited on the etched Ti-6Al-4V plates. The etched surface of the titanium alloy was rough with irregular micro pits—an effect of the HF etching process [37]. Reports suggest that chemical treatment in HF may enhance implant attachment and improve bone-to-implant contact [38]. Thanks to the chemical reaction between titanium (Ti) and hydrofluoric acid (HF) a uniform textured surface is formed across the entire sample [39]. In our study, the average area EDS chemical analysis of the etched titanium alloy plate without the ceramic coating confirmed the substrate alloy composition as consisting of 88.8% titanium, 5.7% vanadium, and 5.5% aluminum (Figure 4a–c).

The pure chitosan coating deposited on the Ti-Al-V alloy was generally smooth, the surface was homogeneous and continuous with a few small visible agglomerates and pores in the whole studied area. The average EDS chemical analysis confirmed the synthesis of the chitosan coating which consisted of carbon, nitrogen, and oxygen (Figure 4d–f).

**Figure 5:**
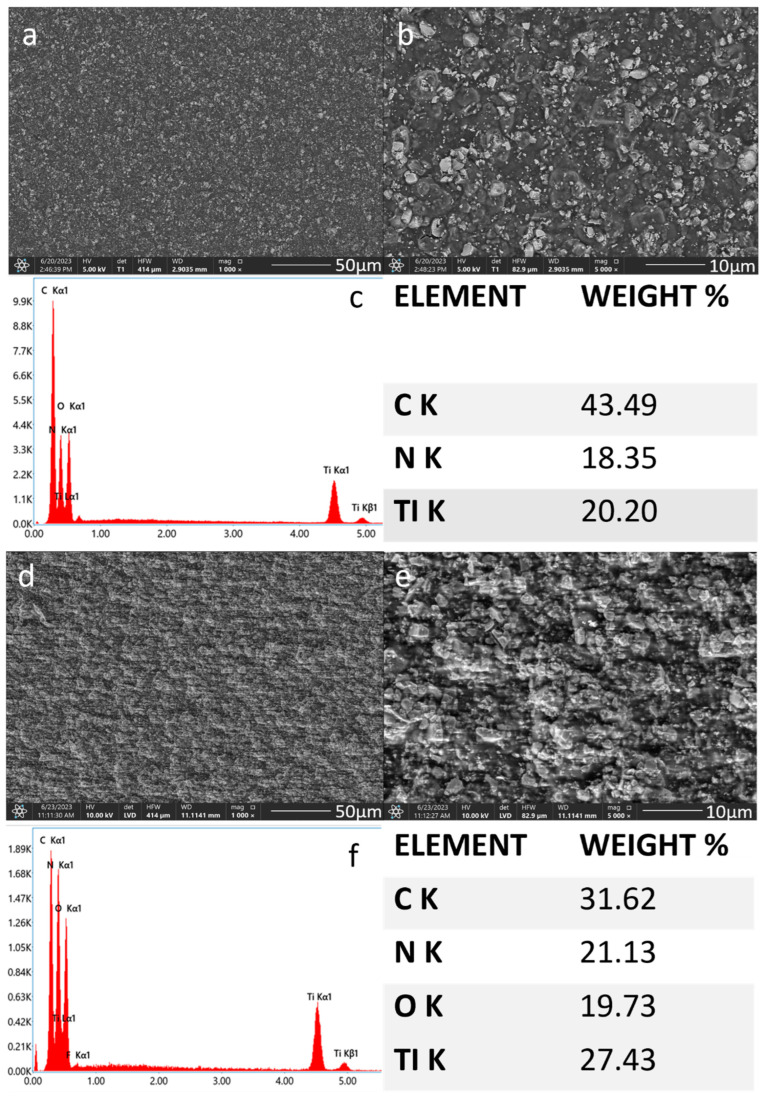
SEM images and EDS spectra with element content of the titanium alloy (Ti-Al-V) etched with HF and covered with chitosan/TiN EPD coatings Ti-Al-V/ch/0.5TiN (**a**–**c**); Ti-Al-V/ch/1TiN (**d**–**f**).

**Figure 6:**
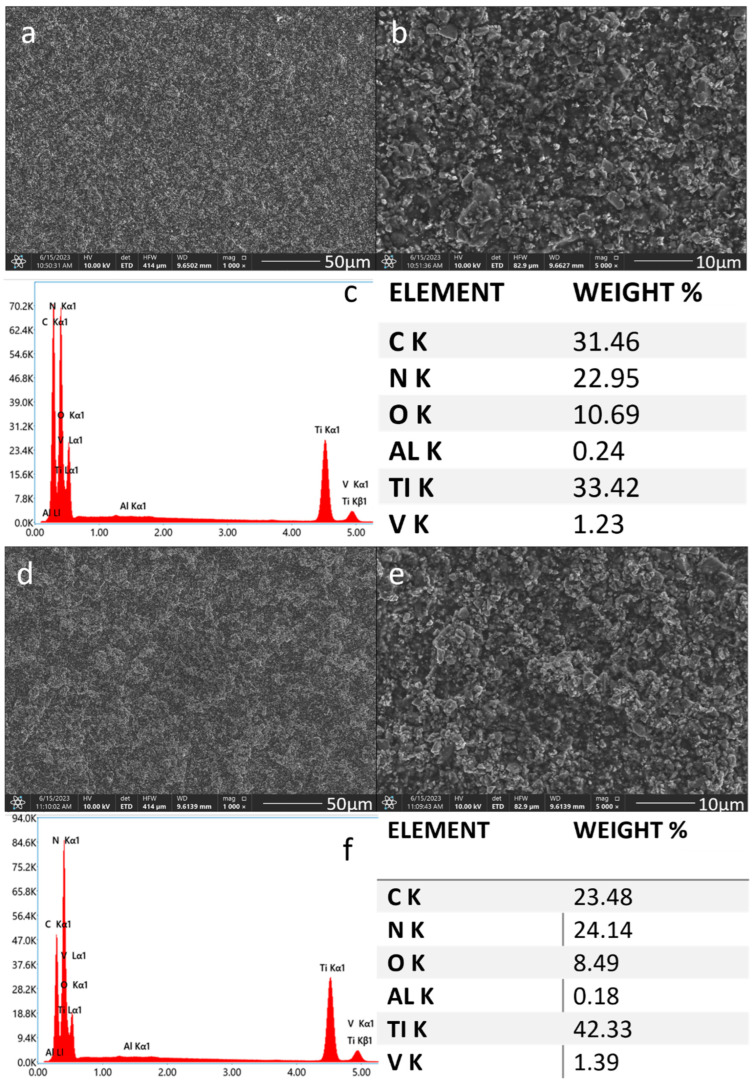
SEM images and EDS spectra with element content of the titanium alloy (Ti-Al-V) etched with HF and covered with chitosan/TiN EPD coatings Ti-Al-V/ch/1.5TiN (**a**–**c**); Ti-Al-V/ch/2TiN (**d**–**f**).

The SEM observations of the chitosan-TiN coatings confirmed the presence of ceramic particles on the Ti-Al-V alloy surface. The SEM images taken at low magnification proved the coatings were continuous and crack-free. As the TiN particles concentration increased, the corresponding increase in the resulting layers density was observed. The average area EDS chemical analysis confirmed the presence of both the chitosan polymer and the nitride particles in the deposited coatings. The average area EDS chemical analysis confirmed the presence of both the chitosan polymer and the nitride particles in the deposited coatings. The EDS analysis showed an increase in titanium and nitrogen weight concentrations in the observed samples, which was consistent with the higher TiN particles concentration in the EPD solution (Figure 5 and Figure 6).

**Figure 7:**
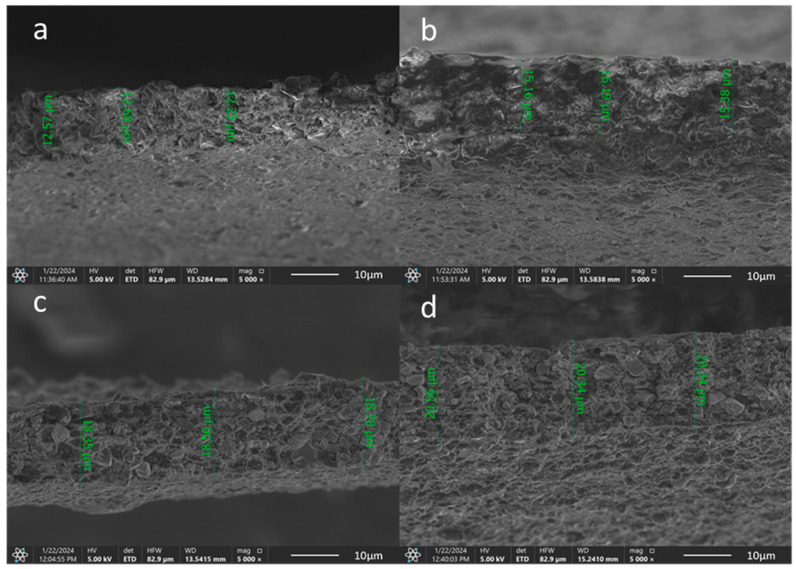
SEM images of the EPD coatings Ti-Al-V/ch/0.5TiN (**a**); Ti-Al-V/ch/1TiN (**b**); Ti-Al-V/ch/1.5TiN (**c**); Ti-Al-V/ch/2TiN (**d**).

The deposited coatings thickness values are presented in Figure 7. The electrophoretically deposited coatings were thicker as the TiN concentration rose in the applied suspension. The values ranged from 12.25 to 20.56 μm.

**Figure 8:**
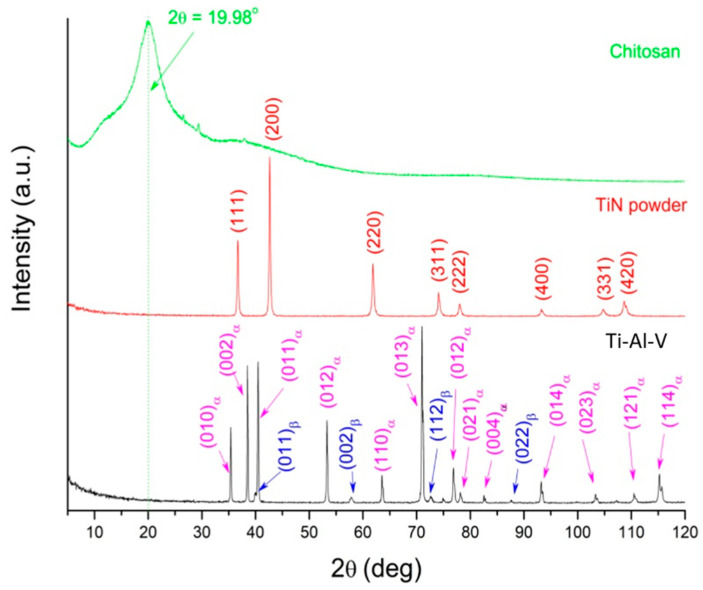
XRD patterns of the Ti-Al-V—matrix, TiN powder and chitosan.

The diffractograms for the composite components, in their initial state, were measured in the classical Bragg–Brentano geometry (Figure 8). The analysis revealed that chitosan existed in an amorphous form. This fact was evidenced by the broadened maximum at the 2θ position at about 20° [28] (Figure 8—green line). The titanium nitride powder was crystalline (Figure 8—red line). The Ti-Al-V substrate turned out to be a two-phase alloy, consisting of an α-phase with a hexagonal crystal lattice and a β-phase—cubic crystal lattice (Figure 8—black line). The comparison of the diffraction line intensities of both phases proved the α-phase to be the dominant one.

**Figure 9:**
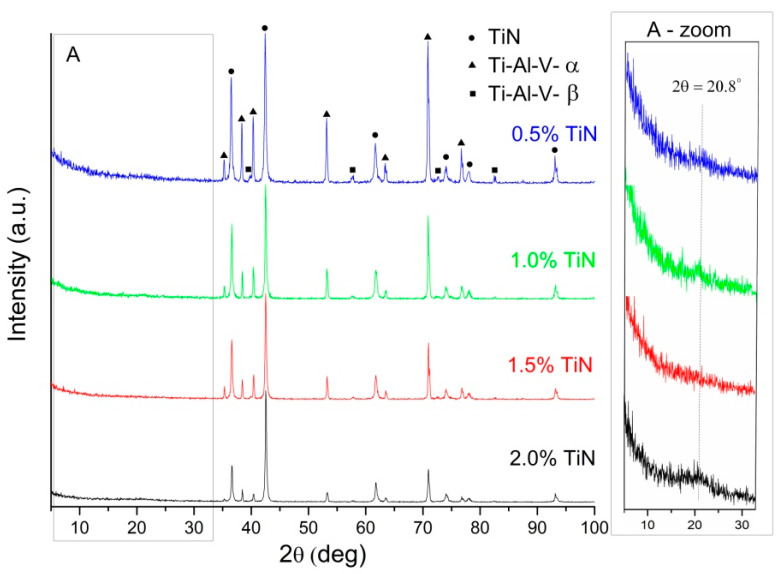
XRD patterns of the Ti-Al-V alloy covered with chitosan/TiN EPD coatings measured in Bragg–Brentano geometry.

Regardless of the TiN content in the mixture used for the layer’s deposition, all the measured diffractograms for the Ti-Al-V substrates coated with the chitosan/TiN layer showed the presence of peaks originating from TiN and the Ti-Al-V-alpha substrate. Moreover, at the 2θ position, around 20.8°, a low-intensity peak appeared, indicating the chitosan presence (Figure 9—A-zoom area). The X-ray examinations confirmed previously discussed results received from the microscopic observations. From the basics of X-ray phase analysis, it has been known that the intensity of diffraction lines is proportional to the amount of phase contained in the mixture. The measured diffractograms in the Bragg–Brentano geometry contain lines belonging primarily to TiN and the alpha phase of the Ti-Al-V alloy (substrate). With the increased content of TiN in the deposited mixture with chitosan, the intensity of the diffraction lines coming from the substrate decreases compared to the TiN’s. It means that the volume fraction of the alpha phase of the Ti-Al-V alloy (substrate) decreases with the volume fraction of the TiN. On the other hand, this fact confirms that the layer thickness increases with the increase of TiN in the deposited mixture.

**Figure 10:**
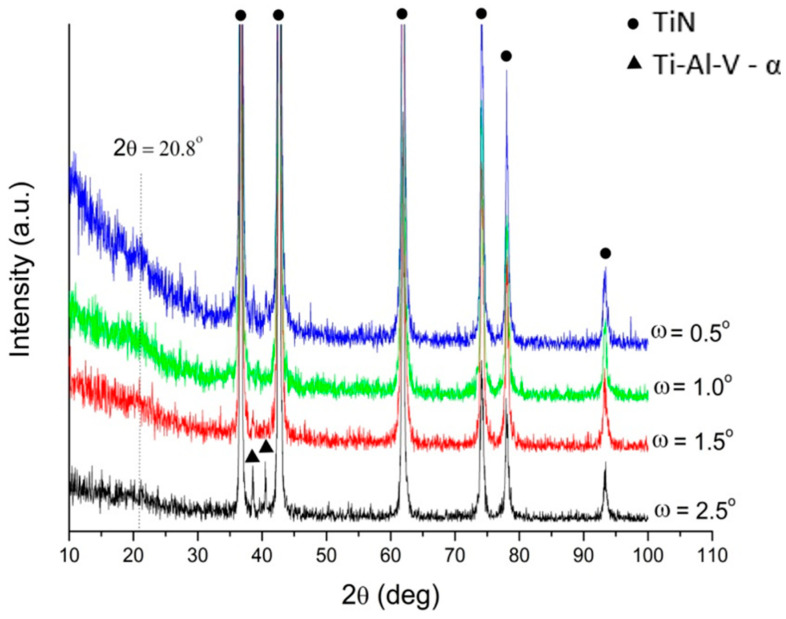
GIXD patterns for the Ti-Al-V/ch/1TiN sample measured at a constant ω angle of 0.5°; 1.0°; 1.5° and 2.5°.

The detailed XRD analysis of the deposited layer was carried out based on diffractograms measured via the grazing incident X-ray diffraction technique (GIXD). The diffractograms were registered at a constant ω angle: 0.5°, 1.0°, 1.5°, and 2.5°. An example of the results received for the Ti-Al-V/ch/1TiN sample is presented in Figure 10. Maintaining a constant angle of the beam during the entire measurement provided a constant penetration depth of X-ray radiation. By reducing the ω angle, structural information was obtained from the increasing depths and finally, from the layer itself. The diffractogram, measured at the ω angle of 2.5 degrees, showed the presence of the two strongest lines derived from the α-phase. The remaining diffraction lines were characteristic of TiN. The diffractograms measured at angles 1.5°, 1.0°, and 0.5° contained diffraction lines from titanium nitride, with a characteristic low-intensity peak originating from chitosan. Similar results were obtained for the remaining titanium nitride contents in the deposit mixture. They confirmed the assumed phase of the layer’s composition.

**Figure 11:**
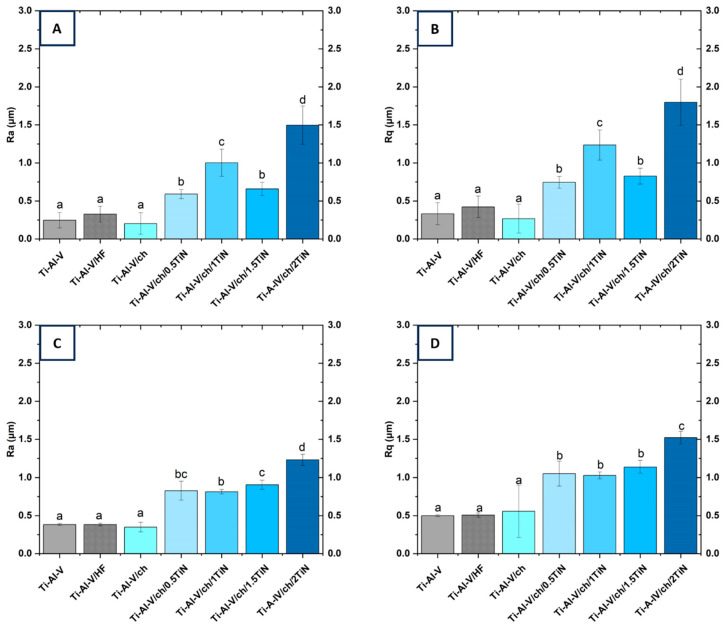
Surface roughness parameters of the titanium alloy (Ti-Al-V) covered with chitosan/TiN EPD coatings, measured via confocal microscopy Ra (**A**); and Rq (**B**) and contact profilometer Ra (**C**); Rq (**D**). Distinct lower-case letters indicate statistically significant differences (*p* < 0.05) in the analyzed parameters among various coating types. Bars labelled with identical letters do not exhibit statistically significant distinctions.

The surface linear roughness parameters, measured by confocal microscopy, are shown in Figure 11A,B. It can be clearly observed that the process of Ti-Al-V alloy etching, prior to the deposition process, increased the surface microroughness, as assessed with the Ra and Rq parameters [39,40,41,42]. Basically, the etching process using hydrofluoric acid (HF) aims to remove adverse surface impurities [38] but it also produces an irregular complex texture that enhances mechanical interlocking between substrate and deposited coatings [39,43]. In our research, the pure chitosan EPD layer, deposited on the Ti-Al-V alloy, significantly enhanced its smoothness, as compared to the etched Ti-Al-V substrate [41,44,45].

The TiN particle’s introduction enhanced the surface roughness in all the tested layers, compared to the pure chitosan layer. The highest linear roughness parameters were observed for the 2 wt.% TiN coating. The heightened roughness resulting from interconnected valleys and peaks on the sample surface may offer benefits for implants, impacting biological and mechanical properties, such as cell attachment, tissue adhesion, and long-term stability [44,46].

**Figure 12:**
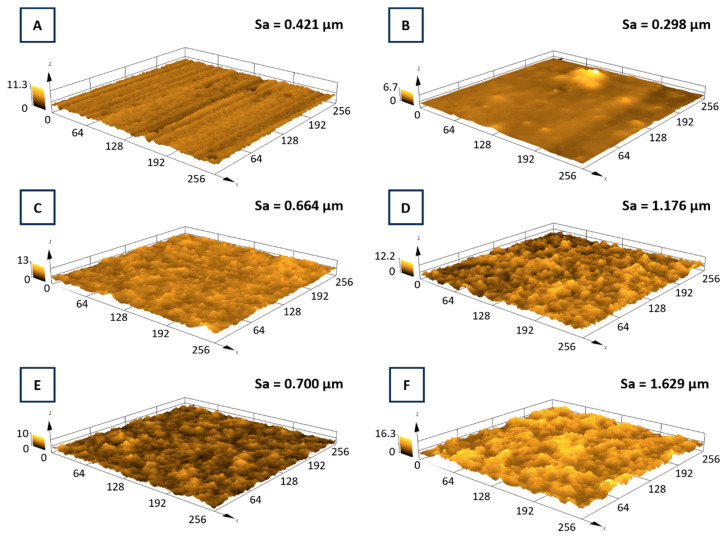
Three-dimensional confocal microscope microphotographs and area roughness parameters (Sa) of titanium alloy (Ti-Al-V) covered with chitosan/TiN EPD coatings. Ti-Al-V/HF (**A**); Ti-Al-V/ch (**B**); Ti-Al-V/ch/0.5TiN (**C**); Ti-Al-V/ch/1TiN (**D**); Ti-Al-V/ch/1.5TiN (**E**); Ti-Al-V/ch/2TiN (**F**). The scale in the charts is described in μm.

The three-dimensional confocal microscope images, as well as the roughness parameter Sa, are in line with the already mentioned linear parameters (Figure 12). The etched Ti-Al-V alloy revealed its linear topography characterized by the presence of linear, parallel grooves likely resulting from the alloy sheets production process. Comparing the micrographs of the etched medical titanium alloy with the pure chitosan layer substrate, there was a significant increase in the surface smoothness in the latter one. The pure chitosan deposition on etched alloy changed the area roughness of the substrate from 0.421 μm to 0.298 μm. With the particle’s gradual introduction into the deposited layers, the surface became more irregular with numerous depressions and thickenings, which was also reflected in the Sa parameters values. The highest area roughness parameter Sa corresponded to the coating deposited with the solution containing 2 wt.% of TiN.

The linear roughness parameters Ra and Rq, as measured via contact profilometry (Figure 11C,D), generally aligned with the results obtained using confocal microscopy; however, slight differences were observed.

It is well known that the surface wettability of bone implants plays a crucial role in achieving a successful biological response [44,47]. This characteristic is reported to exert substantial effects on various cellular processes, including cell attachment [48,49], proliferation [50], and behavior [51,52]. Furthermore, the implant surface wettability significantly influences both tissue integration [43] and protein absorption dynamics [53]. Surface wettability is measured via water contact angles (WCA). Surfaces exhibiting WCA values below 90° are designated as hydrophilic, while those surpassing 90° are categorized as hydrophobic [54,55]. The elevated wettability of implant surfaces is associated with positive outcomes, particularly in terms of enhanced cell adhesion, cell growth, and osteoconductivity [44]. Conversely, surfaces displaying high hydrophilicity may exert detrimental effects on critical protein attachment and escalate thrombogenicity [48].

**Figure 13:**
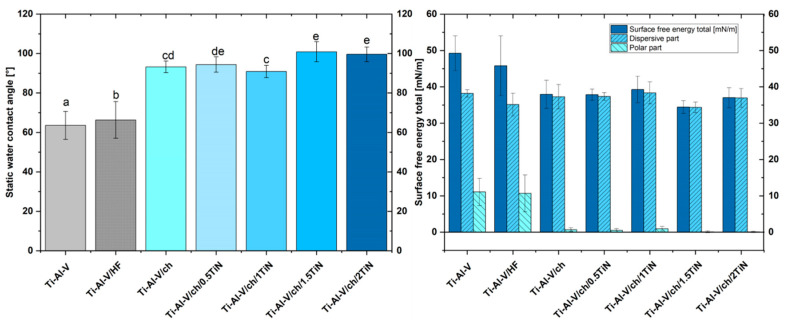
Static water contact angle (**left**) and surface free energy (**right**) of titanium alloy (Ti-Al-V) covered with chitosan/TiN EPD coatings. Distinct lower-case letters indicate statistically significant differences (*p* < 0.05) in the analyzed parameters among various coating types. Bars labelled with identical letters do not exhibit statistically significant distinctions.

**Figure 14:**
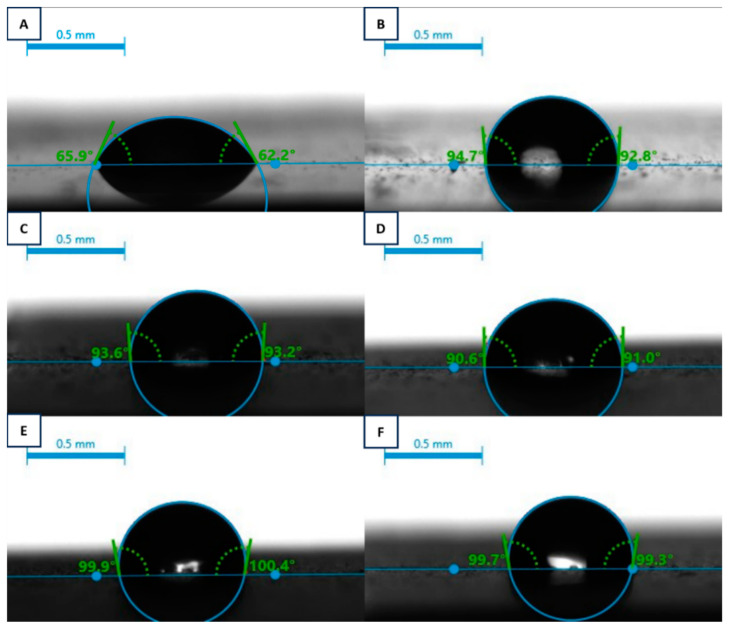
Static Water Contact angle of titanium alloy (Ti-Al-V) covered with chitosan/TiN EPD coatings: Ti-Al-V/HF (**A**); Ti-Al-V/ch (**B**); Ti-Al-V/ch/0.5TiN (**C**); Ti-Al-V/ch/1TiN (**D**); Ti-Al-V/ch/1.5TiN (**E**); Ti-Al-V/ch/2TiN (**F**).

The static contact angles of the chitosan/TiN coatings are shown in Figure 13 (left) and Figure 14. The average contact angle value for the pure Ti-Al-V alloy did not exceed 64°, which indicated the hydrophilic character of the substrate and stood in line with data from the literature [54,56]. The modification of the Ti-Al-V alloy through the etching process slightly increased the static water contact angle of the studied surface [42,57].

Coating the Ti-Al-V alloy with the pure chitosan EPD layer improved the static water contact (from 66° to 93°) which correlated with the tendency mentioned in the literature [44,58]. Simultaneously, the surface free energy decreased from 45.8 to 37.94 mN/m.

The TiN introduction in the deposited layer elevated the static contact angle for all the particles concentrations, when compared to both the pure titanium alloy and the coating containing chitosan polyelectrolyte. The more hydrophobic character of the coatings (Figure 14) containing ceramic particles is most likely connected with the enhanced surface roughness and is supported by Wenzel [54,59] and Cassie-Baxter [60,61] theories describing a relationship between surface roughness and wettability. Surface free energy of the studied samples stands in line with the contact angle results.

**Figure 15:**
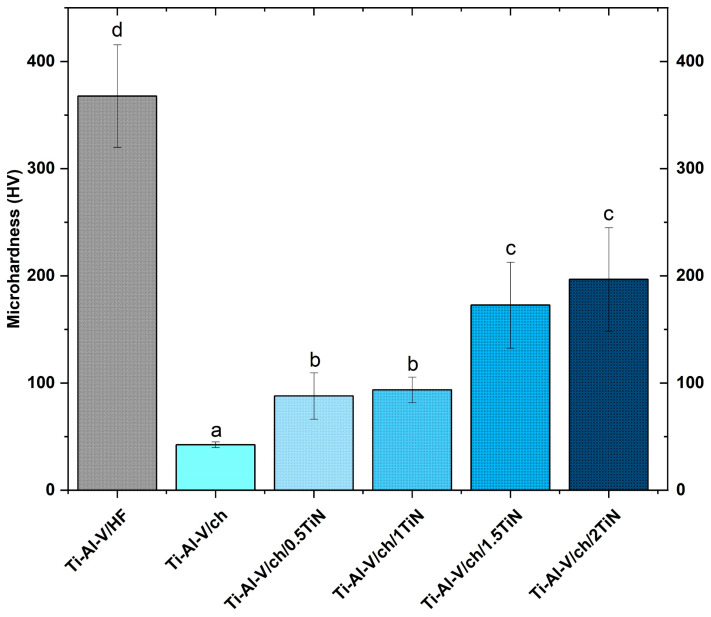
Microhardness of titanium alloy (Ti-Al-V) covered with chitosan/TiN EPD coatings. Distinct lower-case letters indicate statistically significant differences (*p* < 0.05) in the analyzed parameters among various coating types. Bars labelled with identical letters do not exhibit statistically significant distinctions.

The results of the microhardness, measured via the Vickers method, are presented in Figure 15. The etched surface of the Ti-Al-V alloy substrate exhibited the highest hardness among all the tested samples. The measured substrate microhardness value surpassed 360 HV, aligning closely with the data reported in the literature [62,63]. Electrophoretically deposited coatings based on the chitosan matrix significantly lowered the sample hardness across all the TiN concentration variants in comparison to the pure titanium alloy. Comparable patterns were noted in the literature studies [58,64], arising from the distinct material group characteristics, such as chemical bonds [64]. Metals exhibit superior mechanical properties when contrasted with polymers, consequently resulting in lower microhardness measured in coatings containing chitosan polyelectrolyte [64]. The microhardness of the pure chitosan layer was measured at 42 HV, and the value closely aligned with literature data [65].

Microhardness of the coatings containing titanium nitride particles increased with the rising ceramic particles concentrations. The layer containing chitosan and 2 wt.% TiN particles was characterized by the highest microhardness among all the tested EPD coatings.

The determination of the adhesion between the substrate and coating is one of the main concerns when designing layers for medical implants. A strong bond between the coating and the implant surface ensures a durable and reliable connection, influencing the efficiency and long-term functioning of the implant. Strengthening the coating adhesion contributes to minimizing the risk of implant loosening and improves the overall structural integrity. There are numerous available methods to evaluate a coating–substrate attachment. Qualitative methods employ abrasion tests, bend and scratch tests, scotch tape tests, or even X-ray diffraction. Quantitative methods include scratch tests, laser spallation tests, indentation tests, or direct pull-off methods [66].

**Figure 16:**
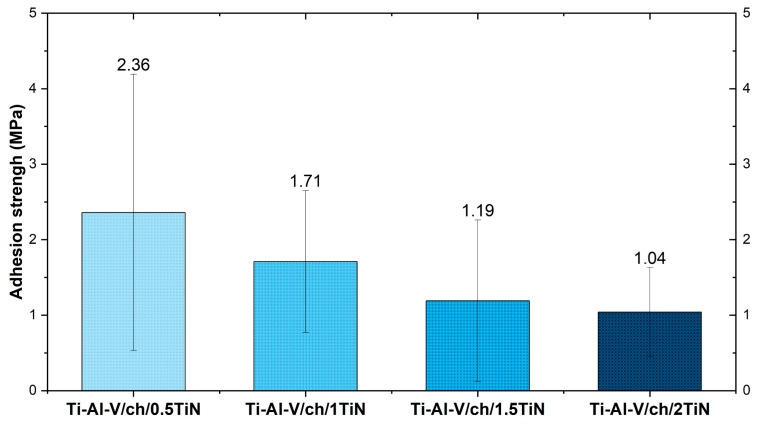
Adhesion strength of titanium alloy (Ti-Al-V) covered with chitosan/TiN EPD coatings [77].

The results of the chitosan/TiN coatings adhesion tests are presented in Figure 16. In our study the coating adhesion was evaluated via the direct pull of method, using the Zwick-Roller testing machine, according to EN 4624.ZP2. Adhesion strength exhibited a decreasing trend with the increase in the titanium nitride particles content in the coating composition. Such a phenomenon is in line with the theory that the adhesion strength decreases with increasing the coating thickness [67]. However, taking into consideration the measurement statistics, the obtained results did not exhibit significant differences depending on the TiN concentration.

The adhesion strength values obtained in our study were comparable to those reported for HAp coatings with similar orthopedic applications, as investigated by Sopcak et al. [68] in the standard tensile adhesion test ISO 13779-4 [69]. However, the relatively modest adhesion strengths of the TiN EPD coatings obtained in our study prompted further investigation into methods to enhance the substrate–coating bonding.

Despite numerous advantages attributed to the EPD coating technique, the adhesion strength of the deposited layers is often insufficient and necessitates post-heat treatment. The underlying principle of this approach is to diminish the free surface energy of the particles by promoting their mutual adhesion [70].

According to Rocha et al. [71], elevated temperatures increase crystallinity and develop chemical bonds between the coating and substrate, contributing to robust coating adhesion. Furthermore, higher temperatures facilitate the formation of chemical bonds across a broader atomic zone, creating a well-mixed coating–substrate interface [71,72].

Moskalewicz et al. [73] used the scratch test to evaluate the adhesion strength of heat treated (390 °C) TiN/chitosan/PEEK layers, electrophoretically deposited onto Ti-6Al-4V alloy. The obtained results were indicative of very good scratch resistance. In the micro-scratch tests, no cohesive cracks or adhesive failures were observed along the scratch track, even under the maximum applied load [73]. Similarly, Zamharrir et al. [70] used the Rockwell C indentation test to assess the adhesion strength of homogenic TiN layers applied via EPD. The obtained results classified their coatings as HF1 grade. In the mentioned work the EPD coatings were sintered at 1200 °C for 2 and 4 h after deposition [70].

In the two mentioned studies the electrophoretically deposited TiN coatings were thermally treated after the deposition process, which might have supported their satisfactory adhesion strength.

The coating–substrate adhesion was also enhanced through the application of a multi-composition gradient or bonding intermediate layer, such as TiO_2_/TiN [66,74,75]. Research findings suggest that the adhesion strength of TiN coating increases in the presence of an additional intermediate TiO_2_ layer between the coating and the surface. The roles of this intermediate layer encompass aiding in effective bonding, stress relaxation, and distribution modification, offering support, enhancing the chemical stability of the substrate, and deepening the hardening depth [66,76].

In summary, while our study found the adhesion strength values of the TiN EPD coatings comparable to those of the HAp coatings in orthopedic applications, further research is needed to enhance the substrate–coating attachment. Elevated temperatures have been shown to improve coating crystallinity and chemical bonding, contributing to stronger adhesion. Additionally, promising results of the heat-treated TiN coatings and studies on multi-composition gradient coatings suggest potential space for improvement. Continued research into optimizing coating deposition methods and post-treatment processes is crucial for enhancing the performance of EPD coatings in orthopedic applications.

## 4. Conclusions

The findings of this study lead to the conclusion that depositing homogeneous EPD coatings of TiN particles and chitosan alters the tribological, physical, and chemical properties of the implant surfaces up to various degrees. In terms of the implant applications, such changes may prove to be desirable. The SEM observations confirmed the homogeneous coating of TiN particles for each tested concentration. As the concentration of TiN particles rose, the corresponding increase in thickness and density of the resulting layer was observed. The XRD analysis confirmed that the deposited layers consisted of the mixed amorphous chitosan and crystalline titanium nitride phases. The contact angle studies revealed that the chitosan/TiN coating deposited onto Ti-6Al-4V substrates changed its surface wettability character from hydrophilic to hydrophobic. The layers containing TiN particles were characterized by higher roughness, as compared to the pure titanium Ti-Al-V alloy. The layer with the highest concentration of ceramic particles (2 wt.%) exhibited the most pronounced surface roughness measured via confocal microscopy and profilometry. The measured microhardness of the deposited layers increased with the rising ceramic particles concentrations in the coating composition. The highest hardness was achieved for the coating containing 2 wt.% of TiN particles, reaching 197 HV. The adhesion tensile strengths of the tested layers decreased with the higher particles content from 2.36 MPa (0.5 wt.% TiN) to 1.04 MPa (2 wt.% TiN). Such values might still not be satisfactory enough as protective layers for implants. Yet, the obtained results are bases for further research on enhancing the substrate–coating adhesion. The deposited coatings demonstrate significant potential for further biological tests regarding their application in veterinary medicine or dentistry.

## Figures and Tables

**Table 1:** Samples nomenclature.

Sample Characteristic	Sample Nomenclature
Titanium alloy Ti-Al-V	Ti-Al-V
Titanium alloy Ti-Al-V etched with 5%HF	Ti-Al-V/HF
Titanium alloy Ti-Al-V with coating containing pure chitosan	Ti-Al-V/ch
Titanium alloy Ti-Al-V with coating containing chitosan and 0.5 wt.% TiN	Ti-Al-V/ch/0.5TiN
Titanium alloy Ti-Al-V with coating containing chitosan and 1.0 wt.% TiN	Ti-Al-V/ch/1TiN
Titanium alloy Ti-Al-V with coating containing chitosan and 1.5 wt.% TiN	Ti-Al-V/ch/1.5TiN
Titanium alloy Ti-Al-V with coating containing chitosan and 2.0 wt.% TiN	Ti-Al-V/ch/2TiN

## Data Availability

The raw data supporting the conclusions of this article will be made available by the authors on request.
